# Application of 3^2^ factorial design for loratadine-loaded nanosponge in topical gel formulation: comprehensive in-vitro and ex vivo evaluations

**DOI:** 10.1038/s41598-024-55953-2

**Published:** 2024-03-16

**Authors:** Durgaramani Sivadasan, Krishnaraju Venkatesan, Jamal Moideen Muthu Mohamed, Saud Alqahtani, Yahya I. Asiri, Mennatullah M. Faisal, Adel Ehab Ibrahim, Yahya Bin Abdullah Alrashdi, Farid Menaa, Sami El Deeb

**Affiliations:** 1 Department of Pharmaceutics, College of Pharmacy, Jizan University, Jizan, Saudi Arabia; 2 Department of Pharmacology, College of Pharmacy, King Khalid University, Abha, Asir Saudi Arabia; 3 Faculty of Pharmacy & BioMedical Sciences, MAHSA University, Bandar Saujana Putra, 42610 Jenjarom, Selangor Malaysia; 4 Department of Pharmaceutics, Faculty of Pharmacy, Zagazig University, Zagazig, 44519 Egypt; 5 Natural and Medical Sciences Research Center, University of Nizwa, Birkat Al Mauz, Nizwa, 616 Oman; 6 College of Pharmacy and Nursing, University of Nizwa, Birkat Al Mauz, Nizwa, 616 Oman; 7 Departments of Medicine and Nanomedicine, Fluorotronics, Inc, and California Innovations Corporation, San Diego, CA 92037 USA; 8 Institute of Medicinal and Pharmaceutical Chemistry, Technische Universitaet Braunschweig, Braunschweig, Germany

**Keywords:** Nanosponge, Loratadine, Ethyl cellulose, Hydrogel, Full factorial design, Sustained release, Drug discovery, Nanoscience and technology

## Abstract

Loratadine (LoR) is a highly lipophilic and practically insoluble in water, hence having a low oral bioavailability. As it is formulated as topical gel, it competitively binds with the receptors, thus reducing the side-effects. The objective of this study was to prepare LoR loaded nanosponge (LoR-NS) in gel for topical delivery. Nine different formulations of emulsion were prepared by solvent evaporation method with polyvinyl alcohol (PVA), ethyl cellulose (EC), and dichloromethane (DCM). Based on 3^2^ Full Factorial Design (FFD), optimization was carried out by varying the concentration of LOR:EC ratio and stirring rate. The preparations were subjected for the evaluation of particle size (PS), in vitro release, zeta potential (ZP) and entrapment efficiency (EE). The results revealed that the NS dispersion was nanosized with sustained release profiles and significant PS. The optimised formulation was formulated and incorporated into carbopol 934P hydrogel. The formulation was then examined to surface morphological characterizations using scanning electron microscopy (SEM) which depicted spherical NS. Stability studies, undertaken for 2 months at 40 ± 2 °C/75 ± 5% RH, concluded to the stability of the formulation. The formulation did not cause skin irritation. Therefore, the prepared NS hydrogel proved to be a promising applicant for LoR as a novel drug delivery system (NDDS) for safe, sustained and controlled topical application.

## Introduction

Allergies arise from an immune system response to a foreign body. Allergic reaction is common and is characterized by the release of histamine, cytokines, and other mediators that may induce mast-cell degranulation. Antihistamines provide symptomatic relief to allergic conditions. Anti-histaminic drugs include loratadine, cetirizine, meclizine, bromopheniramine^1^.

Tricyclic piperidine derivative loratadine (LoR) is a member of the second generation of H_1_-antihistamines and is used to treat urticaria, angioedema, and other allergic skin symptoms. It prevents the symptoms associated with histamines on gastrointestinal smooth muscle, bronchial smooth muscle, capillaries including increased capillary permeability, vasodilation. It is administrated by oral route for the treatment of skin conditions categorized by localized allergic reaction^2^. The cutaneous route should be a better option for drug delivery because the oral route has a limited bioavailability and causes side effects. Because of its high lipophilicity (log p = 5.2) and low molecular weight (382.88 Da), LoR is an excellent option for cutaneous distribution. However, LoR's limited topical applicability is due to its low water solubility^3^.

Nanosponges (NS) are a new class of nanoparticles (NPs) with a small mesh-like 3D structure, smaller than 100 nm, with wide nanometric cavities that encapsulate a wide range of hydrophilic and lipophilic substances. Because of the inner hydrophobic cavities and external hydrophilic branching, they can easily load the particles^4^. The backbone is represented by a long chain of polyesters. The polymers are bound together by small molecules named as cross-linkers. NS can be used to carry water insoluble drugs which mainly belongs to BCS class II and IV^5^. NS are non-irritating, non-mutagenic, non-toxic, non-allergenic and biodegradable which are advantageous compared to other nanoparticulate systems; also, their release is predictable, are stable up to 130 °C and at pH of about 1–11; they can entrap a wide variety of ingredients, protecting the drug from degradation, and offering reduced side effects; they have overall better physical, chemical and thermal stability; they allow extended release for up to 12 h; eventually, these preparations are cost effective^6^.

Hydrogels are cross-linked water-soluble polymers with 3D structure. If the molecular entanglement and/or secondary forces like hydrogen bond, ionic bond are responsible for the formation of linkage, then the hydrogel can be termed as reversible or physical gels. The hydrogels can be prepared by various physical forms like microparticles, slab, coating, films. The loading of the drug in the matrix and the drug release rate also depends upon the porosity of the gel matrix^7,8^.

The novelty of NS hydrogel lies in its unique properties as a highly porous material with nanoscale dimensions that can absorb and retain large quantities of substances such as drugs, toxins, or heavy metals. It can also release these substances in a controlled manner, making it a promising candidate for drug delivery, wound healing, and environmental remediation applications^9^. Additionally, the biocompatibility and biodegradability of NS hydrogels make them a safer and more sustainable alternative to other materials currently used in similar applications.

Pharmaceutical formulations including NPs, NS, liposomes are prepared by employing specific processes that consider a number of different variables and aspects. These independent factors interact to generate efficacy, utility, stability, and safety. As a result, in order to get the intended result, it is frequently required to adjust the formulation processing settings. The quantitative methods intricacy in building the design represents the real link between the contributing elements and reactions, and the detailed for one property isn't necessarily the best for the others^10^. It is common knowledge that traditional experimentation takes a significant amount of time and effort, particularly when evaluating complicated systems.

A full factorial experiment includes every possible level for every component. There are total of 2 k experiments in order to analyse k components at 2 levels. The 2 k full factorial design is quite useful in the early stages of experimental work, particularly in cases when there are less than or equal to 4 process parameters, design parameters, or other components^11^. For factors at 2-levels, it assumes that the response is approximately linear over the range of the chosen factor setting. There are merely two variables in the first design of the 2 k series, A and B, which will each be investigated twice.

**Figure 1:**
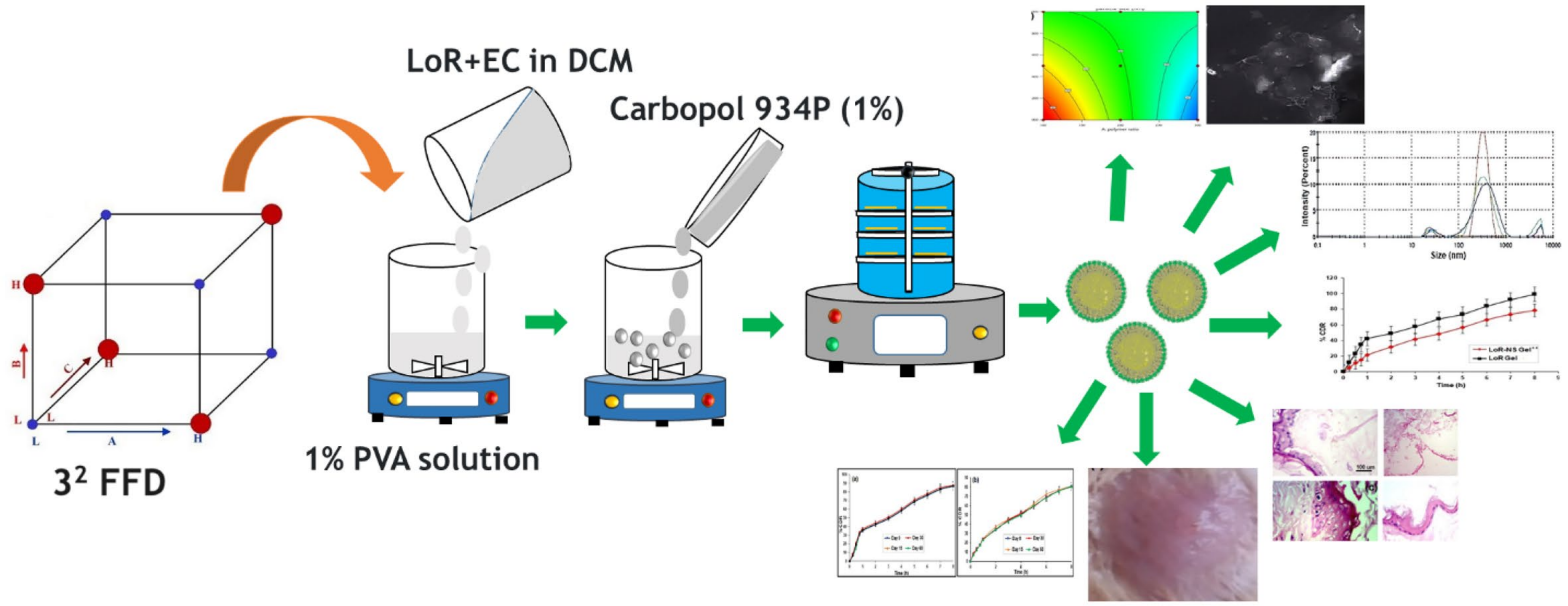
Schematic explanations of the synthesis of NS and its applications.

The current study used the emulsion solvent evaporation approach to create LoR-loaded NS (LoR-NS) in gel. To optimise a 3^2^ Full Factorial Design (FFD), the concentration of the LoR:EC ratio and the stirring rate were changed (Fig. 1). The chosen formula was also examined for cutaneous irritation and histopathology.

## Materials and methods

### Materials

Loratadine (LoR) was a generous gift sample from Apotex Research Pvt Ltd, Bengaluru. Ethyl cellulose (EC) was purchased from Yarrow Chem (Mumbai, India), Dichloromethane (DCM) and poly vinyl alcohol (PVA), HPLC grade water was purchased from SD Fine Chemicals (Bengaluru, India), The carbopol 934P was purchased from Rolex Chemicals (Bengaluru, India). The remaining chemicals and reagents were all analytical grade.

### Optimization and preparation of LoR-NS

LoR-NS formulations were prepared by emulsion solvent evaporation method. The preparation of the NS involves two different phases namely—aqueous phase and dispersed phase (Table 1). The dispersed phase consisting 100 mg of LoR and ethyl cellulose being dispersed in 20 mL DCM^12^. The aqueous phase includes (continuous phase) 1% PVA solution. The dispersed phase was dropped slowly to the aqueous phase which was stirred at a constant rate for 2 h using mechanical stirrer (IKA RW 20 digital, Germany) and the prepared solution containing LoR-NS were collected filtered and dried in oven for 40 °C for one day (Fig. 1).

**Table 1:** Formulation chart of LoR-NS.

Formulation code	LoR:EC ratio (mg)	PVA (% w/v)	DCM (mL)	Distilled Water (mL)	Stirring rate (rpm)
S1	1:1	1	20	100	1000
S2	1:2				1000
S3	1:3				1000
S4	1:1				1500
S5	1:2				1500
S6	1:3				1500
S7	1:1				2000
S8	1:2				2000
S9	1:3				2000

The prepared LoR-NS were fitted into the statistical factorial designs. A 3^2^ FFD was used to examine the impact of the LoR:EC ratio (X_1_) and stirring rate (X_2_) as independent variables on the prepared formulations in vitro properties. LoR:EC ratios of 1:1, 1:2 and 1:3 (X_1_; w/w), and 1000, 1500, 2000 rpm stirring rates (X_2_) were tested. Nine formulations were produced by the combination of the three levels of each variable. The PS (Y_1_), in vitro drug release rate (Y_2_) and ZP (Y_3_) were dependent variables analysed using software from Design-Expert 12 (Stat-Ease) (Table 2). The optimized formulation was selected based on the higher desirability factor. The rationale of selecting control factor was to obtain a desired PS with an in vitro drug release in the NS preparation. To ascertain the degree of significance of the independent factors on the response variables and their interaction, an analysis of variance, or ANOVA, was utilised.

**Table 2:** Levels and factors used in 3^2^ FFD.

Factors	Levels		
	− 1	0	1
LoR:EC ratio, w/w (X_1_)	1:1	1:2	1:3
Stirring rate, rpm (X_2_)	1000	1500	2000

#### Preparation of LoR-NS gel

Hydrogel was prepared by using 1% carbopol 934P (gelling agent). Specified quantity of carbopol 934P was permitted to swell in 100 mL of double distilled water. Approximately 15 mL of gel was added to the optimized NS dispersion by 1%. About 0.02 g of methyl paraben was added for preservative action, the pH of the preparation was adjusted by using triethanolamine. It was continuously stirred at a constant rate for 10 min to allow the formation of carbopol hydrogel integrating LoR-NS^13^. In order to release any trapped air, the produced hydrogel was left undisturbed for 15 min before being stored in a tightly-sealed wide-mouth container for further studies.

### Particle size (PS) polydispersibility index (PDI) and zeta potential (ZP)

The mean PS, ZP of NS dispersion was determined by Dynamic light scattering (DLS) technique using Malvern Zeta sizer Nano S-90 (UK). The dilutions were done using HPLC water. Each measurement was done 3 times based to the technique explained by Mohamed et al.^14^.

### In vitro release study

The in vitro diffusion of LoR-NS was studied using the dialysis bag method. The dialysis membrane-50 (molecular weight- 12 kDa) was saturated overnight in the pH 7.4 phosphate buffer solution (PBS). The donor compartment consists of 5 mL of the NS dispersion being filled in the dialysis bag. The receptor compartment consists of 200 mL of pH 7.4 PBS taken in 250 mL beaker. The beaker was positioned over a magnetic stirrer, and 37 ± 0.5 °C and 100 rpm were maintained as the study state constant^15^. At predefined intervals (i.e., 0.25, 0.5, 0.75, 1, 2, 3, 4, 5, 6, 7 and 8 h), samples (1 mL) were removed and replaced with equivalent volumes of new PBS. The samples were examined for drug concentration using a UV–Vis spectrophotometer set to 250 nm after being suitably diluted. Each and every experiment was run in triplicate.

1 $$\left(\%\right)\mathrm{EE }= \frac{\mathrm{quantity\, of\, LoR\, in\, NCs}}{\mathrm{quantity\, of\, LoR\, in\, the\, preparation}} \times 100$$

### Scanning electron microscopy

The optimized NS dispersion was investigated for surface morphology using SEM (Tescan VEGA3) at different magnifications at room temperature (RT) according to our previous studies^14^.

### Spreadability study

The spreadability was determined by method of horizontal plate glass. A similar weight (2 g) was attached to the upper glass plate, and between two horizontal glass plates about 1 g of the optimized LoR-NS gel was mounted. It had been held upright. It was noticed the duration it took for the top glass plate to separate from the bottom glass plate. The formula below was used to determine the spreadability^16^:

2 $$S = \frac{{\text{M}}\times {\text{L}}}{{\text{T}}}$$

where, S = spreadability; L = length of glass slide (cm), M = weight of the upper slide (g), and T = time taken (sec).

### pH and viscosity determination

A Digital pH metre 335 was used to measure the pH of LoR-NS gel. Prior to analysis, standard buffer solutions with pH values of 4.0, 7.0, and 9.2 were used to calibrate the pH metre. Following calibration, 50 g of gel was submerged in the glass electrode, and the pH was recorded. If any minor pH variations were detected, the pH was brought to skin pH by adding triethanolamine solution dropwise^17^. Using a Brookfield viscometer, the optimised LoR-NS gel's viscosity was determined. The gel was kept at a temperature of 25 °C. After being fastened to the viscometer, Helipath T-bar Spindle No. 95F was submerged in the 50 g gel. Viscosity was measured in centipoises (cps) using a viscometer run at different rpms.

### Drug content

1 g of the gel, precisely weighed, was added to a 100 mL volumetric flask holding 20 mL of PBS (pH 7.4). After 30 min of shaking, 100 mL of PBS pH 7.4 solution was added to the volumetric flask. The sample was examined using an Agilent Technologies Cary 60 UV–Vis spectrophotometer set to 250 nm after an appropriate dilution.

### Ex vivo permeation study

Male Wistar albino rats in good health, weighing between 200 and 250 g, were purchased from an animal shelter in Chennai, India, and housed at 25 °C with a 12-h light/dark cycle. Ad libitum water and regular laboratory pellet diet were provided to the animals. The institutional animal ethical committee (Chairman/ Member secretary of IAEC (Institutional Animal Ethical Committee) and CPCSEA (Committee for the Purpose of Control and Supervision of Experiments on Animals) nominee from K.M. college of Pharmacy, Madurai, India with KMCP/IAEC/8/2020–2021 gave its approval to this study methodology. All animals used in the study were handled with humane care in compliance with the Indian National Science Academy Guidelines for the use and care of experimental animals in research and all the animal experiments were carried out as per CCSEA guidelines. Further, the experimental data are reported as per the ARRIVE guidelines 2.0 checklist of information.

The ex vivo permeation study of the optimised LoR-NS (test) and the pure LoR (control) plain gel were studied. The rats were sacrificed by cervical dislocation (generally approved as minimal suffering for small rodents) and the dorsal skin was shaved and removed. The skin was divided for test and control. The skin was then attached to the Franz diffusion cell. The stratum corneum faced the donor compartment, while the dermis faced the receptor compartment, due to the manner it was linked. To the donor compartment, about 5 g of the gel for test and control was applied. The receptor compartment was filled with PBS (pH 7.4, mimicking the pH of blood). Subsequently, the beaker was kept on the magnetic stirrer (100 rpm) with temperature of 37 ± 0.5 °C. At predefined intervals (i.e., 0.25, 0.5, 0.75, 1, 2, 3, 4, 5, 6, 7 and 8 h), the samples (1 mL) were removed and replaced with an equivalent amount of freshly prepared buffer. The samples were diluted appropriately, and then their drug concentration was measured using a UV spectrophotometer set at 250 nm. The data were fit into different kinetic models^18^. The permeation of LoR-NS gel across the skin was calculated using the following formula:

$$J = \frac{{\text{V}}}{{\text{A}}} \times \frac{{\text{dc}}}{{\text{dt}}}$$

where, J = flux; A = surface area; V = receptor volume; C = concentration; t = time.

### Histopathological study

After performing the ex vivo permeation study, the excised skin was stored in 10% formalin. Then, the skin was dehydrated using ethanol and embedded using paraffin blocks. Hematoxylin and eosin (H&E) was used for further staining, after that, these stained tissues were examined using a light microscope fitted with a digital camera system. Pictures were taken, and these were then analysed^19^.

### Skin irritation study

A study on skin irritation was conducted on six rats (N = 6) of either sex and 200–250 g in weight. The animals were kept in cages made of polypropylene and had free access to water and a conventional meal. There were two groups of animals (n = 3 per group). The dorsal region hair follicles were removed with an operating blade and around 4 cm of scissors. Gel was used and wrapped with a cotton bandage after the hair was removed^20^. The following number method was used to score the reaction at the application site after it was examined:

- Formation of eschars and erythema: 0 indicates no erythema, 1 indicates very little erythema, 2 indicates well-defined erythema, and 3 indicates moderate to severe erythema.
- Formation of edoema: 0 indicates no edoema; 1 indicates extremely minor edoema (barely noticeable); 2 indicates slight edoema (area margins well raised); 3 indicates moderate edoema (raised about 1 mm); 4 indicates severe edoema (increased more than 1 mm).
- Initial cutaneous irritation score: This is how the main skin irritation was rated: One (1) is non-irritating (0.0); two (2) is inconsequential (0.1–0.4); three (3) is minor (0.41–1.9); and four (4) is severe (5.0–8.0) irritant.

### Stability study

Stability studies were performed for the optimized LoR-NS gel. It was stored in a wide mouth screw capped container and was placed in the stability chamber (Thermolab, Scientific equipment Ltd, India) maintained at temperature 40 ± 2 °C/75 ± 5% RH for 3 months^13^. This study accelerates the degradation processes, allowing scientists and researchers to assess the product degrades over time in a relatively short period.

### Statistical analysis

The means ± standard deviations were used to present the quantitative data. One-way analysis of variance (ANOVA) was used to conduct statistical comparisons using SPSS 13.0 for Windows software (SPSS, USA). *p*-values were deemed statistically significant if they were less than 0.05.

## Results and discussion

Information on NS drug delivery systems from the past research was carried out using FFD. The researchers reported that factor design plays a very important role in optimizing drug dosage forms; by using the design of optimization, the researchers can carry out the formulations with minimum number of runs and obtain optimizing results^21^. So FFD was employed in the current research. The two independent variable was controlled at the same time and the effects were analyzed either individually or together in the design.

### Optimization of PS, ZP, and drug release

There are three factors has been selected considering the various factor and level. There are LoR:EC ratio, X_1_, and stirring rate, X_2_. The effect of studied variables (LoR:EC ratio, X_1_; stirring rate, X_2_) on the response variables (PS of the NS, Y_1_; release rate, Y_2_; ZP, Y_3_) was statistically analyzed using Design-Expert 12 (Stat-Ease) software. To determine the significance and the interaction between the independent factors of the response variables, the ANOVA was performed. The relationship between independent variable and the response was described by means of polynomial equations. The equation was based on the selected model^22^.

Factors that influence NS quality included PS with long-acting drug release. Combination of optimal level factor which influenced PS with long-acting drug release are LoR:EC ratio at level 1 (1:2), stirring rate at level 2 (1350 rpm).

Coded factors equation:

3 $${\text{Y}}_{1} = + 267 + 10.22*{\text{A}} + 14.07*{\text{B}} + 21*{\text{AB}} - 10.375*{\text{A}}^{2} + 43.63*{\text{B}}^{2}$$

**Figure 2:**
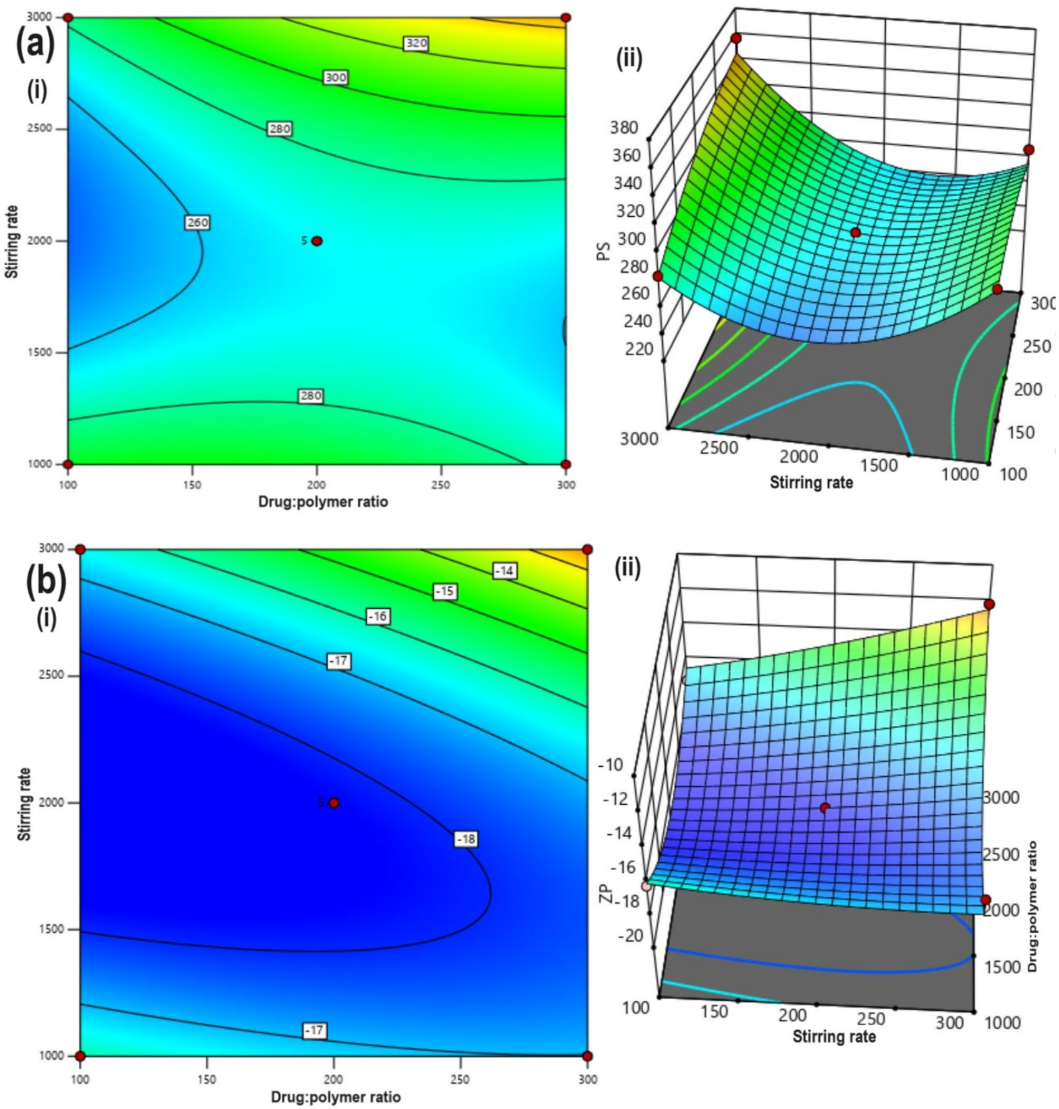
(**a**) PS (**b**) ZP of plots from 3^2^ FFD (i) contour plot and (ii) 3D response surface plots of LoR-NS from various trails; the significant variances associated with control (pure LoR) are denoted by **p > 0.05, as estimated by the ANOVA test.

Table 3 shows the average PS of the prepared LoR-NS, where F = 4.12, *p* > 0.05, R^2^ = adeq. precision = 2.410, and R^2^ = 0.8714, the mean PS varied between 284.23 ± 15.1 and 368.7 ± 74.97 nm with all the formulation exhibited PS in the nanoscale (< 1 μm) range. ANOVA was used for statistical analysis, and the results revealed a linear relationship, showing that the interaction between the two independent variables on the PS of NS was not statistically significant (*p* > 0.05). From the equation, at higher stirring rates (> 1000 rpm), the reduced PS could be attributed to the increased mechanical shear forming smaller NS as shown in contour plot Fig. 2a(i). Accordingly, the emulsion globules aggregate and lead to increased PS at lower stirring rates in the 3D graph Fig. 2a(ii). Collectively, the increase of PS values (formulations S1 and S2) by emulsion globules is most likely due to increase of the LoR:EC concentration along with slow stirring rate (1000 rpm)^23^.

**Table 3:** PS and ZP of LoR-NS.

Formulation	PS (nm)	ZP	Percentage release after 8 h (%)
S1	357.5 ± 14.6	− 12.22 ± 1.6	90.76 ± 55.5
S2	328.6 ± 25.4	− 15.18 ± 1.08	91.75 ± 7.62
S3	368.7 ± 74.97	− 11.45 ± 1.12	90.46 ± 7.54
S4	233.9 ± 10.5	− 18.81 ± 1.62	95.22 ± 6.2
S5	274.7 ± 28.6	− 17.14 ± 1.81	86.2 ± 7.26
S6	284.23 ± 15.1	− 17.22 ± 1.04	92.74 ± 8.55
S7	298.90 ± 14.2	− 16.09 ± 1.06	95.99 ± 8.31
S8	287.9 ± 23.23	− 16.18 ± 1.77	95.13 ± 9.23
S9	285.2 ± 25.2	− 15.82 ± 1.92	95.18 ± 9.47

All the values are expressed as Mean ± SD, n = 3.

Factors that influence NS quality included PS with long-acting drug release. Combination of optimal level factor which influenced PS with long-acting drug release are LoR:EC ratio at level 1 (1:2), stirring rate at level 2 (1350 rpm).

The responses such as ZP and drug release have been found to be insignificant (Fig. 2b(i)). The formula with highest desirability (0.895) was selected as optimized formula and was prepared for further studies. Desirability is a statistical approach used to evaluate and select the most favourable combination of factor levels that simultaneously meet or optimize multiple criteria or responses. The desirability score ranges from 0 to 1, with 1 indicating the most desirable outcome. It takes into account the specified target values or ranges for each response variable and assigns a score based on how well the chosen factor levels meet these criteria^24^. This could include factors such as PS, ZP, and drug release rate that contribute to the overall effectiveness and quality of the formulation.

Coded factors equation:

4 $${\text{Y}}_{{2}} = - {18}.{26} + 0.{7743}*{\text{A}} + {1}.0{26}*{\text{B}} + {1}.{2525}*{\text{AB}} + 0.{27}0{6}*{\text{A}}^{2} + {2}.{51}*{\text{B}}^{2}$$

The linear model assessed the design, and Eq. (4) was established using the chosen model, with Y_2_ representing the response variable (ZP). Analyzing the equation, it was noted that ZP decreased, attributed to the statistically significant negative coefficient value of B (F = 4.21; *p* < 0.05, R^2^ = 0.9809, Adeq Precision = 4.011), coinciding with an increase in the stirring rate, as illustrated in Fig. 2b(ii).

Coded factors equation:

5 $${\text{Y}}_{{3}} = + {91}.{81} - {2}.0{67}*{\text{A}} - {1}.0{7}*{\text{B}}$$

(where F = 4.10, P < 0.05, R^2^ = 0.9907, Adeq Precision = 5.011).

**Figure 3:**
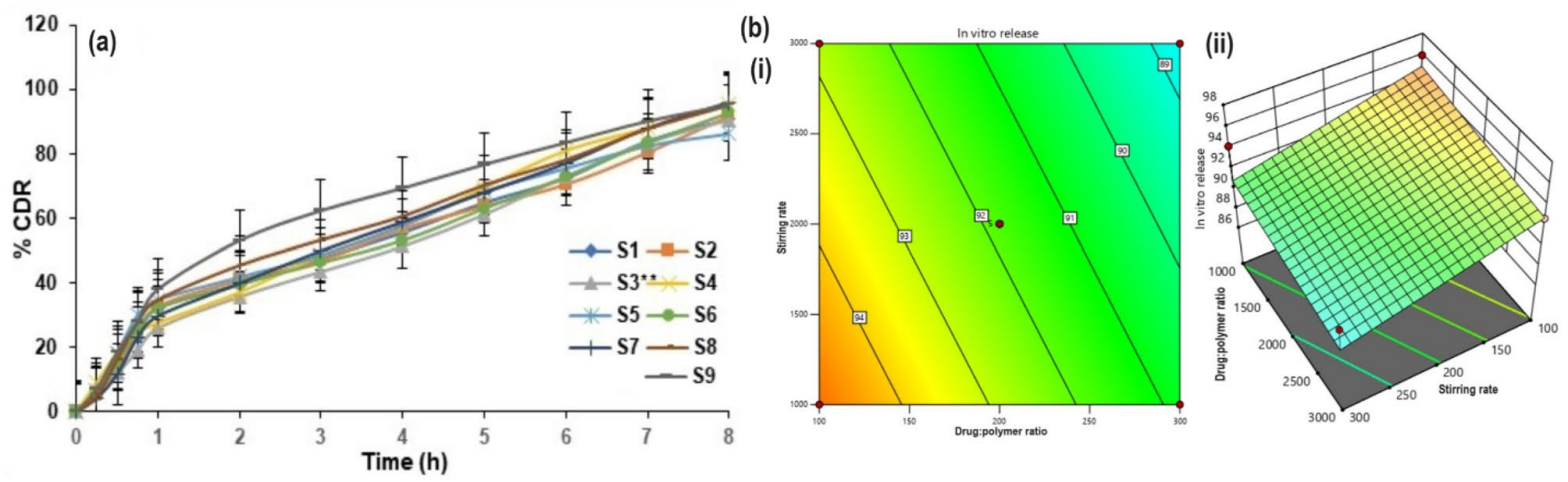
(**a**) Cumulative percentage drug release of LoR-NS from various trails (mean ± SD, n = 3) and (**b**) in vitro drug release plots from 3^2^ FFD (i) contour plot and (ii) 3D response surface plots of LoR-NS from various trails; the significant variances associated with control (pure LoR) are denoted by **p > 0.05, as estimated by the ANOVA test.

The in vitro release profiles of LoR-NS (percentage of cumulative drug release) are illustrated graphically in Fig. 3a. According to Table 3, the proportion of LoR released after eight hours varied from 86.2 ± 7.26% (S5) to 95.99 ± 8.31% (S7). ANOVA was used in the statistical study to compare the release of LoR from various formulations, and the results showed (Fig. 3b(i)) that the release was significant (R^2^ = 0.9976). The increase in the wall thickness of NS may be the cause of the decrease in the LoR % released after 8 h with the rise in the polymer ratio (*p* > 0.05) (Fig. 3b(ii)). This can result in a longer diffusional path which reduces the release of LoR-NS^25^.

It was evident that, the stirring rate increases and subsequently the NS size decreases. This increase could be the result of increased LoR entrapment in the hydrophobic matrix due to decreased porosity, which could have lowered release rates and the similar result was reported by Aldawsari et al. (2015). Likewise, Chabria et al. (2021) investigated that the larger particles with higher porosity would allow hydrogel to leak the drug, resulting in faster release rates. Because of ECs hydrophobic and plastic nature, the particles adjacent to the surface matrix may initially be released resulting in an initial burst effect^26,27^.

### Surface morphology

**Figure 4:**
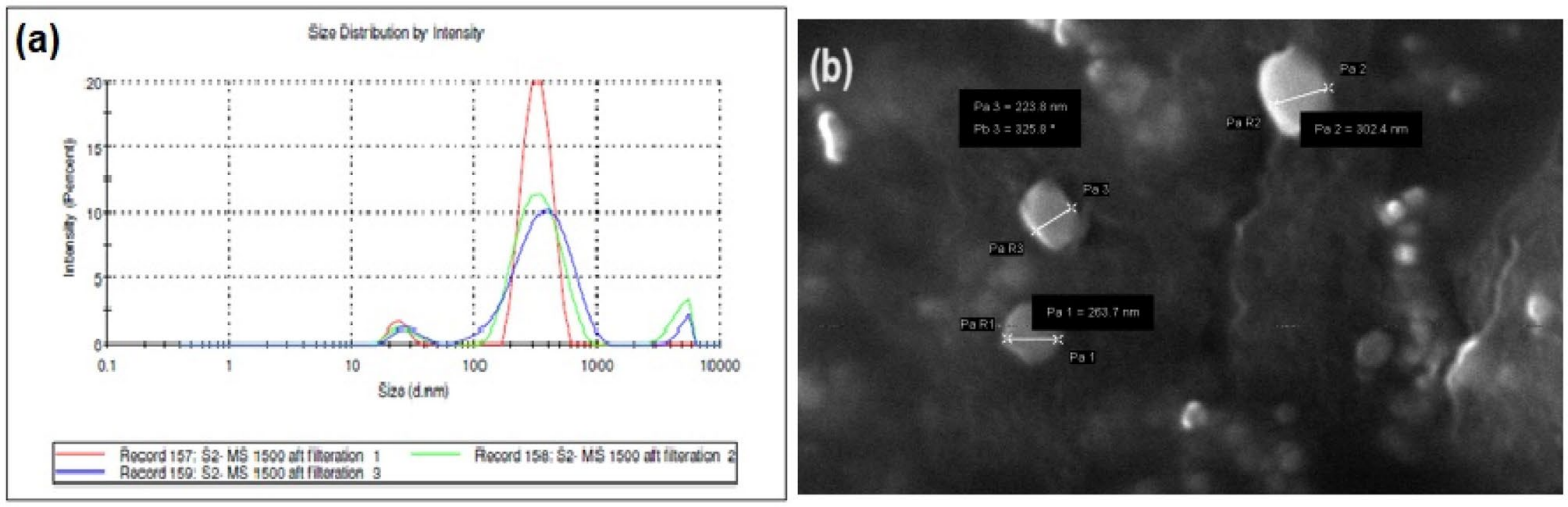
(**a**) PS of optimized formulation and (**b**) SEM images of LoR-NS.

The average diameter was 298 ± 4.45 nm and was determined by counting the more than 450 number of NPs visible in several SEM pictures, and by collecting SEM images of native LoR using the same dispersion and staining methodology as for the LoR dispersion. The NPs and NS have a tendency to aggregate or agglomerate, especially in aqueous solutions (diluted liquid). These aggregates can significantly affect the DLS measurements and result in variations in the PSD. The degree of aggregation leading to differences in the peak positions and intensities^28^. various instrumental factors, including laser intensity and detector sensitivity also responsible for these variations. DLS measurements involve statistical analysis of scattered light intensity fluctuations, and as such, inherent statistical variations can contribute to slight differences in the measured PSD values (Fig. 4a). The images revealed that the particles were spherical in shape, nanometric in size and was found to be porous in nature. It is most likely the result of DCM diffusing from the surface of the NPs during preparation because there are tiny pores on the NS's surface. Certain materials may exhibit surface reconstruction, faceting, or roughness, which can give rise to non-spherical features on the nanoparticle surface (Fig. 4b). To measure the average diameter of LoR-NS, which had previously been sonicated into a dispersion, SEM images were acquired^29^.

### Viscosity, pH, drug content, and spreadability

The spreadability of the optimized LoR-NS gel formulation was found to be 0.65 ± 0.03 g cm/s. Viscosity is an important parameter of the gel because as the viscosity decreases the spreadability also decreases. Viscosity was found to be 75.9 ± 5.63 cps by using T-bar spindle (95F). Drug content was found to be 94.6 ± 6.15% (Table 4).

**Table 4:** Viscosity, pH, Drug content, and spreadability of LoR-NS gel.

Formulation	Viscosity (cps)	pH	Drug content (%)	Spreadability (g cm/s)
LoR-NS gel	75.9 ± 5.63	6.98 ± 0.07	94.6 ± 6.15	0.65 ± 0.03

*All the values are expressed as Mean ± SD, n = 3.

The proper method for pH measurement in high viscosity gel includes conventional pH measurement techniques. The steps are as follows: the selection of the right electrodes, ensuring that the gel is uniformly mixed and free of air bubbles, and maintaining controlled temperature. Specialized electrodes with a gel penetration design are available for samples with high viscosity. These electrodes are designed to penetrate through the gel, allowing for a more accurate measurement. Diluting the gel with an appropriate buffer solution is recommended to reduce viscosity if the gel is too thick for conventional pH measurements. If possible, stir or homogenize the gel during the pH measurement process to temporarily reduce viscosity. This can facilitate better contact between the electrode and the sample. Additionally, consider investigating in-line pH measurement during the production process^30^. The pH was found to be 6.98 ± 0.07, which is close to the pH of the skin.

NS was considered to have a strongly spreadable because of its limited time spread. The therapeutic efficacy of gels is influenced by their distribution. To help ensure that the gel is applied to the skin uniformly, the produced gels must be easily spreadable and satisfy the ideal standards for topical application. Furthermore, this is believed to be a crucial component of patient adherence to treatment^31^. Given that anti-inflammatory and topical analgesic formulations are applied to the thin layers of the skin, the consistency of the material is one of the most crucial components. The gels viscosity is crucial in regulating the penetration of the medication.

### Ex vivo permeation of LoR-NS gel

**Figure 5:**
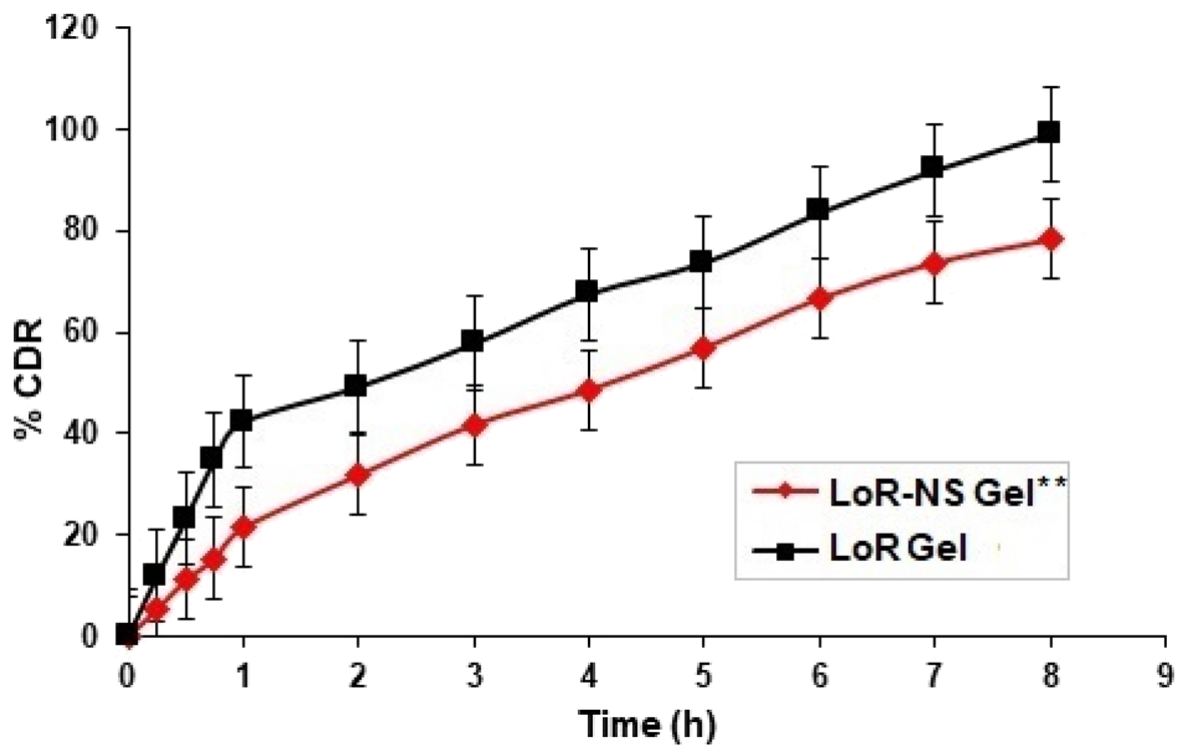
Percentage cumulative drug release profiles of LoR-NS gel and LoR gel. The significance variance associated with control (LoR gel) are denoted by **p > 0.05 as estimated by ANOVA text.

Ex vivo permeability of LoR-NS gel across the skin was found to be 2.266 mg/cm^2^/min. Different models were adopted for fitting the drug release data in kinetic modelling for optimized formulation and revealed that ex vivo permeation study follows zero order kinetics (R^2^ = 0.9752), drug diffused from the matrix framework (Fig. 5). The 'n' value refers to the non-Fickian diffusion of all formulations (n = 0.6618), that indicates that there was absence of limit that splits the medium and the drug as described the Mohamed et al.^32^.

The higher and faster drug release in the control gel compared to the NS gel might be explained by factors such as LoR encapsulation, matrix effects, and differences in size and surface area. The study's adoption of zero-order kinetics aligns with the controlled-release nature of the nanosponge gel, where the release rate remains constant over time, emphasizing its suitability for the drug delivery applications^33^.

Consequently, the amount of LoR increases that permeates the skin. EC included in DCM increase skin permeability by changing or disorganizing the ordered alkyl chains of phospholipids, which causes lipid fluidization^34^. Lipid fluidization increases the LoR ability to penetrate the skin. The lipid bilayers in the stratum corneum are able to penetrate using DCM, which lowers the stratum corneums barrier resistance and increases intracellular transport by dekeratinizing corneocytes.

### Histopathological outcome

**Figure 6:**
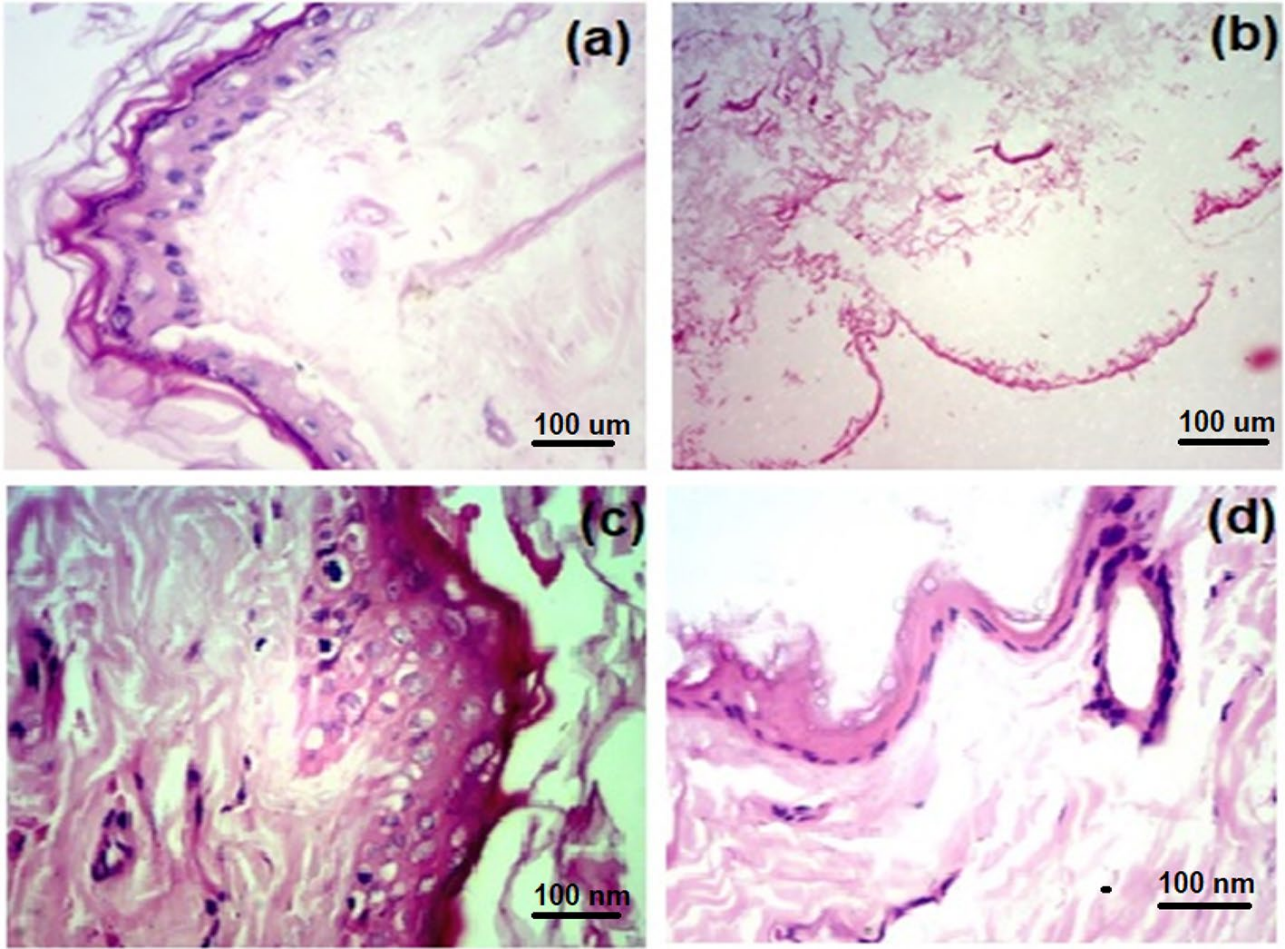
Histopathological reports of (**a**) normal skin (Untreated-control), (**b**) LoR solution, (**c**) LoR-NS suspension, and (**d**) LoR-NS gel.

The potential histopathological changes were examined on the excised skin of rat. The histopathology of the skin exposed to various control, LoR solution, LoR suspension, and LoR-NS gel (Fig. 6). The histoarchitectural presentation of skin revealed intact epidermis and dermal structures. The study revealed intact epidermal layers and dermal structure shown in Fig. 6a. The histoarchitectural presentation of this skin presented a few autolytic changes in a diffused manner (Fig. 6b). No inflammatory infiltrates were observed, nor any degenerative changes were observed in the given tissue section (Fig. 6c,d). The degenerative changes often manifest as alterations in cellular morphology, tissue structure, or function, and their absence in this case is indicative of a well-preserved tissue structure. This finding may imply that the tissue is not undergoing pathological changes associated with aging, metabolic disorders, or other degenerative conditions^35^.

### Skin irritation

**Figure 7:**
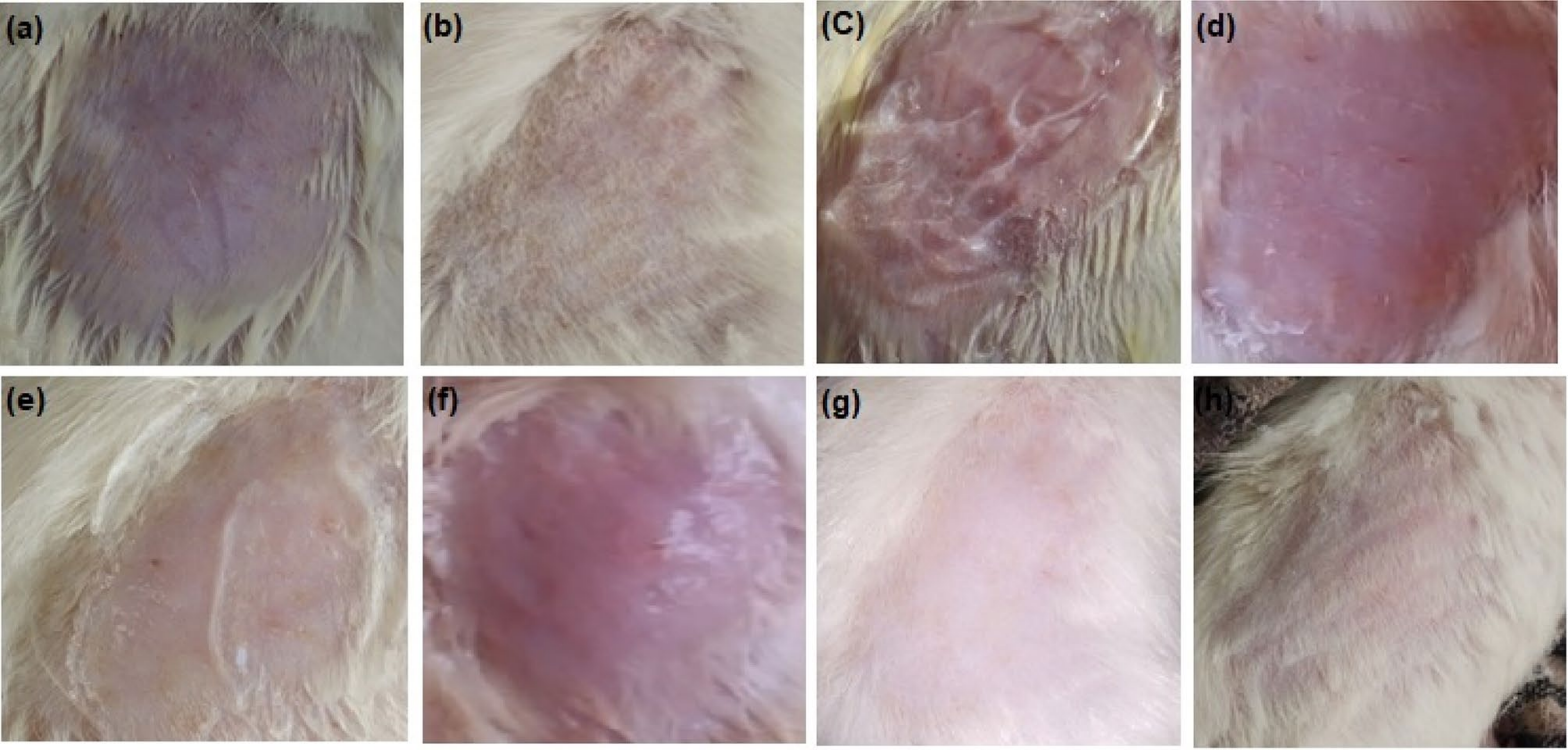
(**a**) control group, (**b**) test group at day 0, (**c**) test group at day 1, (**d**) test group at day 2, (**e**) test group at day 3, (**f**) test group at day 5, (**g**) test group at day 6, and (**h**) test group at day 7.

The skin irritation studies of control and test group up to day 7 illustrates in Fig. 7. The erythemal scores were recorded in control and test group for all the animals. The mean erythemal scores were found to be 0.00, which means that there was no edema or erythema on the skin of the shaved rats in the optimized formulation^36^.

### Stability of LoR-NS gel

The chosen conditions (40 ± 2 °C, 75 ± 5% RH) aim to simulate the potential challenges that the product might face during transportation, warehousing, or storage in regions with varying climates. The elevated temperature and humidity levels help to identify potential issues related to stability in these environments. They often promote the most common degradation mechanisms observed in pharmaceuticals, such as hydrolysis, oxidation, and physical changes. By subjecting the product to these conditions, researchers can assess its stability and make predictions about its shelf life^37^.

**Figure 8:**
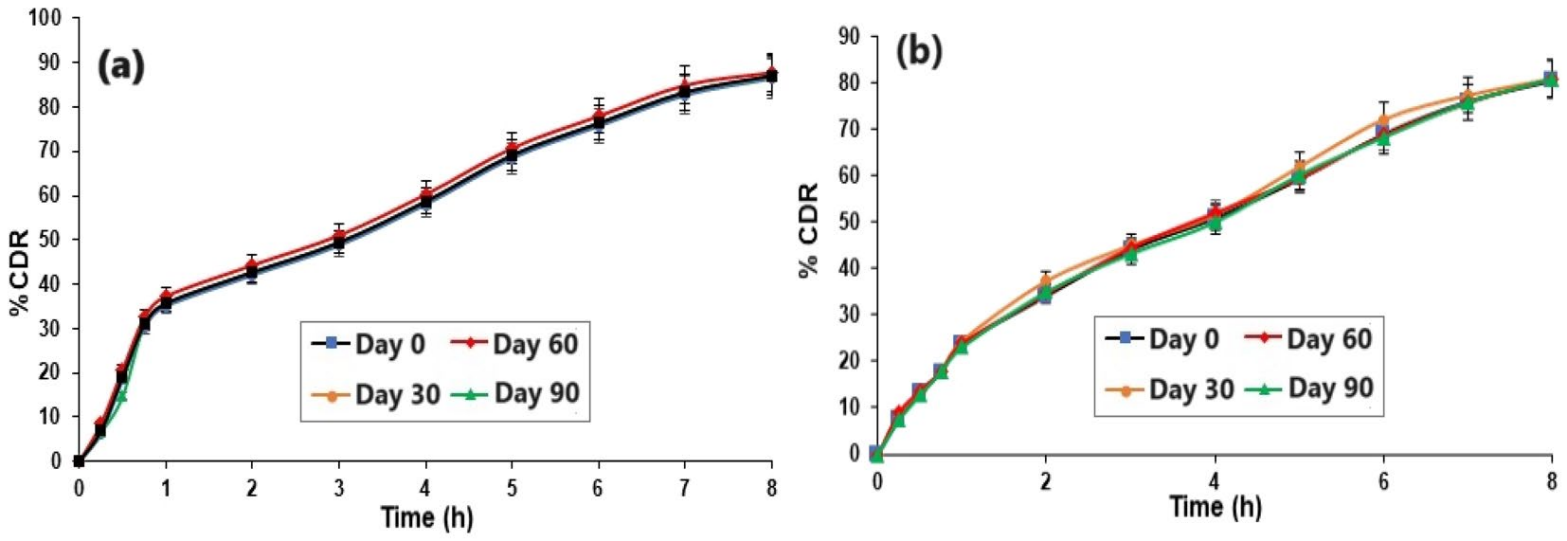
In vitro drug release profile during 8 h in which medium (PBS, pH 7.4) at 40 ± 2 °C, 75 ± 5% RH of (**a**) LoR-NS suspension and (**b**) LoR-NS gel. No significant differences were observed overtime and between (**a**) and (**b**).

The formulations were stable based on drug EE, and cumulative % drug release (CDR %) after their storage for 3 months (90 days) at 40 ± 2 °C, 75 ± 5% RH. Also, the spreadability, viscosity, and pH of LoR-NS gel were stable in the same experimental conditions. The data obtained with LoR-NS suspension corroborate previously published data^38^. There was no significant difference (*p* > 0.05) in stability after 2 months, and the slight changes observed in its pH, %CDR, viscosity and % EE but not significantly difference after 90 days as given in Table 5 and Fig. 8. Mainly pH reduction observation was due to hydrolysis (LoR might undergo hydrolysis usually at high temperatures) or oxidation with chemical reactions and degradation of buffers in the formulations^39^. The stability study conclude that the optimized formulation should remain stable when topically applied in humans.

**Table 5:** Stability profile of LoR-NS gel at 40 ± 2 °C /75 ± 5% RH for 90 days.

Days	LoR-NS suspension		LoR-NS gel			
	Drug EE (%)	CDR (%)	Spreadability (g.cm/sec)	Viscosity (cps)	pH	CDR (%)
0	97.51 ± 3.63	86.42 ± 5.08	0.65 ± 0.03	75.9 ± 3.63	6.98 ± 0.34	79.47 ± 5.13
30	96.60 ± 5.1	86.88 ± 7.05	0.63 ± 0.02	74.8 ± 4.21	6.98 ± 0.88	80.90 ± 4.20
60	98.85 ± 5.03	87.1 ± 5.06	0.67 ± 0.02	71.6 ± 3.14	6.98 ± 0.47	81.87 ± 3.09
90	96.29 ± 8.18	85.37 ± 10.6	0.61 ± 0.45	69.4 ± 5.59	6.48 ± 0.18	78.31 ± 7.16

*All the values are expressed as mean ± SD, n = 3.

## Conclusion

In conclusion, the comprehensive exploration of the Loratadine-loaded nanosponge incorporated into a topical gel through a Complete Factorial Design has yielded promising results through a series of rigorous evaluations. The in vitro studies provided valuable insights into the preparation physicochemical attributes, drug release kinetics, and stability. The *ex-vivo* evaluations, utilizing skin permeation studies, further demonstrated the efficacy of the nanosponge-based gel in facilitating the transdermal delivery of Loratadine. These collective findings highlight the potential of the Loratadine-loaded nanosponge in topical gel as a promising candidate for transdermal drug delivery, with implications for improved therapeutic outcomes and patient compliance. Further studies and clinical trials will be crucial to validate and expand upon these results, paving the way for the potential translation of this innovative formulation into clinical practice.

## Data Availability

The datasets used and/or analysed during the current study are available from the corresponding author on reasonable request.

## Abbreviations

LoR: Loratadine
NS: Nanosponge
LoR–NS: Loratadine loaded nanosponge
PVA: Polyvinyl alcohol
EC: Ethyl cellulose
DCM: Dichloromethane
FFD: Full factorial design
PS: Particle size
ZP: Zeta potential
DLS: Dynamic light scattering
EE: Entrapment efficiency
SEM: Scanning electron microscopy
NDDS: Novel drug delivery system
NPs: Nanoparticles
HPLC: High performance liquid chromatography
PBS: Phosphate buffer solution
Rpm: Revolution per minute
h: Hour
